# A visual representation of cattle movement in Ireland during 2016

**DOI:** 10.1186/s13620-018-0129-x

**Published:** 2018-09-07

**Authors:** Guy McGrath, Jamie A. Tratalos, Simon J. More

**Affiliations:** Centre for Veterinary Epidemiology and Risk Analysis, UCD School of Veterinary Medicine, University College Dublin, Belfield, Dublin, D04 W6F6 Ireland

**Keywords:** Cattle movements, Marts, Biosecurity, Epidemiology, Disease transmission, Geographical information systems, Animal welfare, Risk

## Abstract

The aim of this study was to create a clear visual representation of the live movements of cattle in the Republic of Ireland over the course of the year 2016. The animation created can be viewed online: https://youtu.be/PTCdPMnenBw This animation was created to be a communication tool to enable stakeholders to appreciate the extent of high risk cattle movements (farm to farm, farm to market to farm) in the Republic of Ireland and to highlight the potential role that these movements may play in the spread of infectious diseases of cattle in Ireland from one farm to another.

## Introduction

In Ireland, cattle movement and trade has been a feature of rural life since at least medieval times. Through the centuries, local fairs (*aonach* in Irish) and markets (*margadh*) have played an important role in rural society, with cattle making up the greater part of stock offered for sale [1, 2]. The trade of live animals remains an important component of the cattle industry and, indeed, the farming way of life. More than half of this trade is through markets (‘marts’), the remainder being direct farm to farm sales, some of which are mediated online.

The role of cattle movement in disease spread is well recognised and documented [3–6]. Mee et al. [7] identified a wide range of pathogens that could be introduced through introduction of purchased or leased cattle, including viruses, bacteria and protozoa. These authors highlighted the importance of bioexclusion: preventive measures designed to avoid the introduction of pathogenic infections. In Ireland, important infectious diseases of cattle include bovine tuberculosis, Johne's disease (paratuberculosis), bovine viral diarrhoea (BVD) and infectious bovine rhinotracheitis (IBR). Sayers et al. [8] has identified some of the challenges to biosecurity on Irish farms, including factors that influence appropriate implementation, and Irish information is now available to raise awareness of farm biosecurity and promote good practice [9–12].

There are many country-specific studies of cattle movement, further to the seminal work from the UK [13–16], focusing on movement and contact networks per se [17] and on the application of this information for improved disease surveillance and control [18–20]. Similar work has yet to be conducted in Ireland. Statistical reports on cattle movements are produced annually [21] in the form of summary information, however, these do not illustrate in any detail the spatiotemporal patterns of movements. Here, we have produced an animation representing cattle movements in the Republic of Ireland in 2016. Our broader aim is that this will be used as a communication tool to enable herd owners, veterinarians and other stakeholders to understand the scale and extent of cattle movements, and the roles that these may play in the transmission of disease.

## Methodology

DAFM’s Animal Identification & Movement (AIM) database contains records of all cattle movements in the Republic of Ireland. This animation presents 1.3 million of these movement events (for example, the movement of a trailer - which may contain a number of cattle - from farm to market), consisting of 6.7 million individual cattle movements (a movement from farm to market and subsequent onward movement to farm are counted as two movements in this study). To maintain farm anonymity, herd locations were randomly placed (‘jittered’) on farmland somewhere within 5 km of the location of their largest fragment of land. Due to low numbers and the possibility of identification, farms on islands were removed from the study. All movement events between jittered farms and facilities were calculated from the AIM data. Movement events (and by extension, live animal movements) were allocated one of two risk categories according to their potential to facilitate the spread of infectious diseases of cattle in Ireland from one farm to another via live animal movements: *‘High Risk’* movements, those which concluded in animals being on a farm (shown in red in the animation), including mainly sales via a market and farm to farm sales, but also imports, and *‘Low Risk’* movements, mainly those which resulted in slaughter but also moves to export points (shown in blue). The number of animals per movement event was also recorded and represented as the size of the place marker disc (or ‘dot’) at movement start/finish locations. For the national overview, the movement arcs grow at 5 km per frame, hold for 9 frames and decay over 18 frames – representing a total of approximately 5 days in real-time. For the low altitude scenes, the time is slowed, with the movement arcs growing at 4 km per frame, holding for 50 frames and decaying over 25 frames – representing approximately a day in real-time. Distances are straight-line calculations and are therefore more conservative than true road-network distances. Distances were calculated for imported cattle from the centre of the country of origin but not for live exports which were only calculated to the point of departure from the Republic of Ireland. Imports from Northern Ireland are designated as United Kingdom imports and therefore appear to originate from mainland Great Britain rather than Northern Ireland in the animation.

## Results

**Fig. 1:**
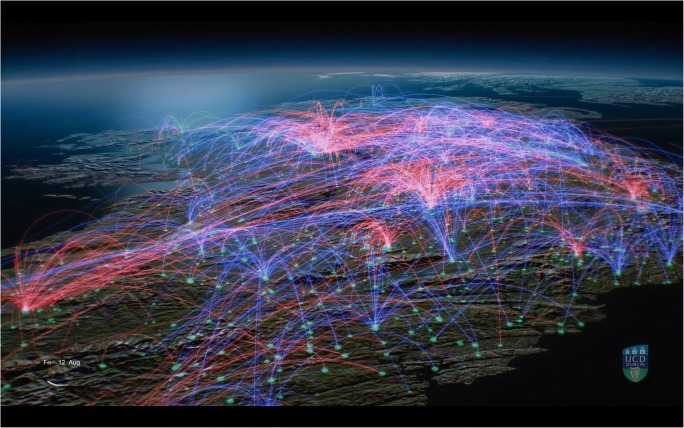
Screenshot of an animation showing cattle movement events (for example, the movement of a trailer, which may contain a number of cattle) in Ireland. Red lines represent ‘High Risk’ moves, those where animals are traded between herds in Ireland (including trade via a market), as well as a small number of imports; blue lines represent ‘Low Risk’ - moves to slaughter or an export point. In this study, a movement from farm to market and subsequent onward movement to farm was counted as two movement events

**Fig. 2:**
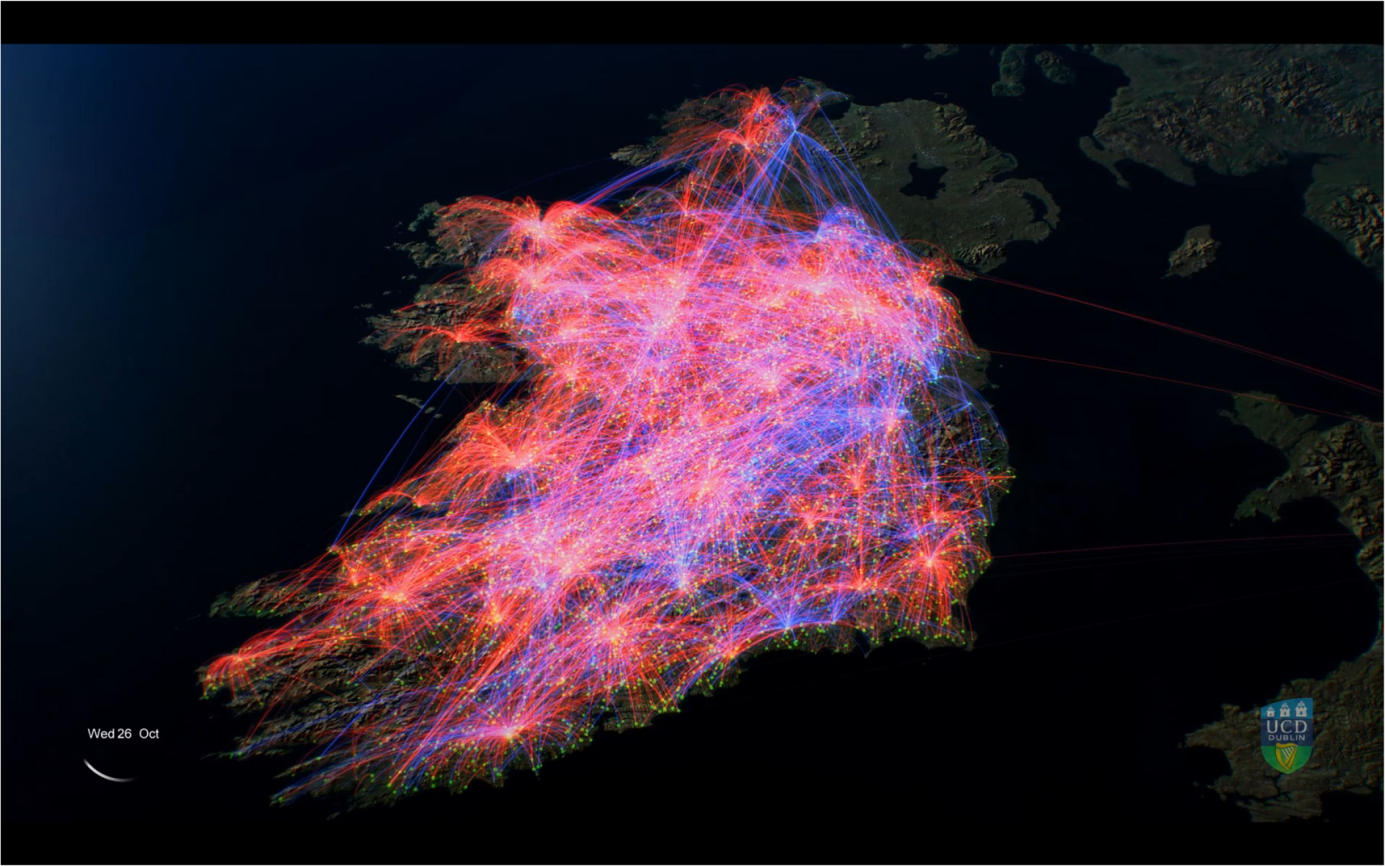
Screenshot of an animation showing cattle movement events (for example, the movement of a trailer, which may contain a number of cattle) in Ireland. Red lines represent ‘High Risk’ moves, those where animals are traded between herds in Ireland (including trade via a market), as well as a small number of imports; blue lines represent ‘Low Risk’ - moves to slaughter or an export point. In this study, a movement from farm to market and subsequent onward movement to farm was counted as two movement events

Figures 1 and 2 show screenshots of the animation, which can be viewed online at https://youtu.be/PTCdPMnenBw.

There were 1,314,365 movement events in 2016, of which 1,020,715 (78%) were ‘High Risk’ and 293,650 (22%) ‘Low Risk’. These represented, respectively, 6,669,022, 4,778,408 (72%) and 1,890,614 (28%) individual animal movements, respectively. Of the ‘High Risk’ movement events, 375,767 (37.0%) were movement events from a herd to a market, 388,360 (38.0%) from a market to a herd, 255,959 (25.1%) from a herd directly to another herd, and 629 (0.06%) of imported animals. These represented 3,511,541 individual animal movements to or from markets, 1,262,596 herd to herd and 4,271 imports. Of the ‘Low Risk’ movement events, 288,975 (98.4%) movement events were to slaughter and 4,675 (1.6%) to export, representing 1,745,044 and 145,570 individual animal movements, respectively.

**Fig. 3:**
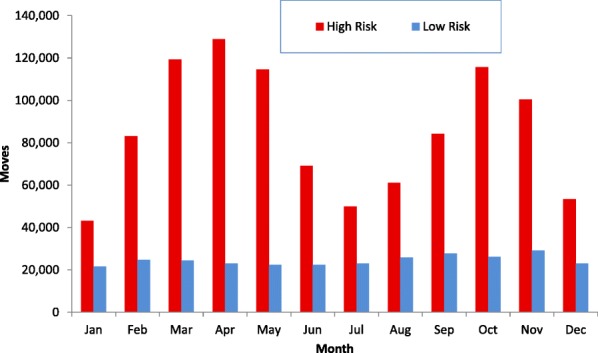
Number of cattle movement events (moves; for example, the movement of a trailer - which may contain a number of cattle - from farm to market) in Ireland, by month, and by risk category, during 2016. ‘High Risk’ moves represent moves where animals are traded between herds in Ireland (including trade via a market), as well as a small number of imports; whereas ‘Low Risk’ moves represent moves to slaughter or an export point. In this study, a movement from farm to market and subsequent onward movement to farm were counted as two movement events

**Fig. 4:**
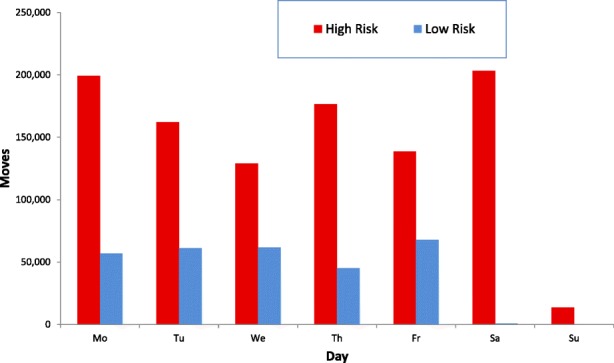
Number of cattle movement events (moves; for example, the movement of a trailer - which may contain a number of cattle - from farm to market)(moves) in Ireland, by day of week and by risk category, during 2016. ‘High Risk’ moves represent moves where animals are traded between herds in Ireland (including trade via a market), as well as a small number of imports; whereas ‘Low Risk’ moves represent moves to slaughter or an export point. In this study, a movement from farm to market and subsequent onward movement to farm were counted as two movement events.

There was considerable seasonal variation in the ‘High Risk’ data, which showed distinctive seasonal peaks in Spring and Autumn, with lows in January, July and December, but relatively little seasonality in the ‘Low Risk’ (Fig. 3). There were in total only 800 ‘Low Risk’ movement events on a Saturday or Sunday, whereas Saturday was one of the busiest days for ‘High Risk’ movement events (Fig. 4).

## Discussion

Limited studies have been done on cattle movements in Ireland, despite the availability of data on all cattle movements and births going back to the year 2000. To date, this has been limited to investigations of movements in a specific area of Ireland [22] or with specific age groups of cattle [23]. Other studies have used movement data as a component of broader investigations into the aetiology or management of specific diseases [24–28], but as yet, there has been no systematic analysis of cattle movements in Ireland as a whole. This is the first study to provide a detailed, and visual, overview of the magnitude and scope of live cattle movement in Ireland.

Substantial movement of cattle in Ireland has long been recognised. With this, and planned subsequent work, we are now able to present information providing quantitative insights into its magnitude. The 1.3 million movement events shown in the video, representing all journeys travelled by vehicles, such as trailers, to transport cattle to markets, new herds, slaughter plants or export facilities in 2016, cover a cumulative distance of 46 million kilometres. That is the equivalent of circumnavigating the Earth 1,015 times or travelling to the moon and back 60 times. Similarly, the cumulative distance travelled by the 6.7 million animals travelling on these same journeys is 283 million kilometres, which is equivalent to the distance to the sun and back. It would take more than 15 min travelling at the speed of light to cover this distance.

Many of the movement events shown in red (moves to and from markets as well as between farms), and which are of most concern in the spread of cattle diseases, appear to pulsate. This is driven by the days on which markets operate, Monday to Saturday (Fig. 4). In these movement events, the points which appear to represent very intense levels of activity are in most cases markets. The concentration of market and farm to farm sales in the spring and autumn, which contrasts with the fairly uniform distribution of low risk disposals, is discernible as the animation progresses over the course of the year. The slow, low altitude scene from January 23rd to January 30th illustrates the variation in movement over the course of a week, the lack of Sunday trading being very evident.

It is clear from this animation that many movements cover very large distances, in some cases almost the entire length or breadth of Ireland. These long-range movements are often to abattoirs, suggesting the existence of relatively high purchase prices attracting herd owners away from facilities closer to home.

It can be difficult to present this information in a manner that is interesting and arresting, both for farmers and the industry more broadly, hence, the decision to visually represent these movement data. Data visualisation is being used extensively to assist farmers with other areas of decision-making, for example, to communicate the likely impact of climate change on land use [29]. We are not aware of earlier similar approaches in this field, and hope that this animation will be a valuable communication tool to enable stakeholders to appreciate the role that live cattle movements may play in the transmission of disease. Further, we hope that this animation will stimulate interest in the spatial and temporal patterns of Irish cattle movements. Work is currently being undertaken to compare Irish cattle movements with those from 11 other European countries, as well as a more detailed examination of the spatiotemporal patterns of movements in Ireland, and their implications for the control and monitoring of diseases of livestock. Further work is also planned on the role of cattle movements in the spread of specific diseases, each highly relevant to Ireland, including paratuberculosis, bovine tuberculosis and bovine viral diarrhoea.
